# Prevalence and Genetic Characterization of Mammalian Orthoreoviruses in Diarrheic Cattle from Guangxi, China

**DOI:** 10.3390/vetsci13030225

**Published:** 2026-02-27

**Authors:** Haonan Yu, Yuhang Luo, Zhen Liao, Li Fan, Haolan Zhong, Kang Ouyang, Ying Chen, Yeshi Yin, Zuzhang Wei, Yifeng Qin, Qingting Dong, Yan Pan, Weijian Huang

**Affiliations:** 1 Laboratory of Animal Infectious Diseases and Molecular Immunology, College of Animal Science and Technology, Guangxi University, Nanning 530004, Chinazuzhangwei@gxu.edu.cn (Z.W.); qinyf@gxu.edu.cn (Y.Q.); 2 School of Animal Science and Technology, Guangxi Vocational University of Agriculture, Nanning 530009, China; 3 Guangxi Key Laboratory of Animal Reproduction, Breeding and Disease Control, Nanning 530004, China; 4 Guangxi Zhuang Autonomous Region Engineering Research Center of Veterinary Biologics, Nanning 530004, China

**Keywords:** bovine diarrhea, L1 RT-PCR, σ1 protein, segment reassortment, temporal phylogenetics

## Abstract

Mammalian orthoreoviruses (MRVs) infect a wide range of animal hosts, yet epidemiological data from southern China remain limited. Here, we performed regional screening of diarrheic cattle in Guangxi, China, from 2023 to 2025. Using L1-targeted RT-PCR, MRV was detected in 8.4% (15/178) of fecal specimens collected across eight prefectures, with a higher detection rate during the cool (autumn–winter) season (October–March). We obtained two complete S1 segments, assigning one Guangxi strain to MRV1 and the other to MRV3. Phylogenetic analysis of L1 sequences indicated a single local lineage nested within a broader multi-host cluster, which was consistent with segment reassortment. σ1 protein alignment revealed a conserved central/C-terminal scaffold and a variable distal head; notably, the MRV3 σ1 sequence contained an extended ~360–390 aa region compared with MRV1. Overall, these findings provide an epidemiological baseline for Guangxi cattle, integrating detection rate, serotype context, and seasonality to support targeted, seasonally informed surveillance and MRV risk assessment.

## Simple Summary

Diarrhea in cattle harms animal welfare and farm income. We set out to learn how common a group of viruses called mammalian orthoreoviruses are in diarrheic cattle in Guangxi, China, and what kinds are present. From 2023 to 2025, we collected 178 stool samples from sick cattle on farms across Guangxi and tested them for these viruses. We found the virus in 15 animals (about eight percent). Detections were more frequent in the cooler months (autumn and winter) than in the warmer months. We read part of the virus genetic code from selected positive samples and found at least two distinct types in Guangxi. When compared with records from other regions, the local viruses were closely related to strains reported in cattle and other animals. Our study provides a clear depiction of this virus in local herds during the study period. The results can help farms and laboratories plan practical testing and routine hygiene measures to manage diarrhea.

## 1. Introduction

Bovine diarrhea remains a major cause of morbidity and production loss, particularly in young calves, and its etiology is typically multifactorial. Several viruses are repeatedly implicated in diarrhoeic outbreaks, including bovine rotavirus, bovine coronavirus, and bovine viral diarrhea virus; however, clinical presentation alone rarely allows for pathogen attribution [1,2,3]. In addition to viral agents, non-viral enteric pathogens—such as *Cryptosporidium* spp., enterotoxigenic *Escherichia coli*, and *Salmonella* spp.—are frequently involved, and mixed infections are common [4,5]. Consequently, surveillance and differential diagnosis that consider the broader enteric disease complex are essential to interpret detection results and to prioritize control measures.

Mammalian orthoreoviruses (MRVs) are non-enveloped, double-stranded RNA viruses (genus *Orthoreovirus*, family Reoviridae) with a 10-segment genome (L1–L3, M1–M3, S1–S4) [6,7,8,9,10]. MRVs have been detected in many mammalian hosts and are most often linked to enteric and respiratory disease, with occasional reports of severe neurological outcomes in humans [11]. The segmented genome enables reassortment during co-infection, which may generate novel genotypes with an altered fitness or host range. MRVs are classically grouped into three serotypes (MRV1–3) based on the S1-encoded σ1 attachment protein, and a fourth serotype has been proposed [6,7]. Although MRV is not routinely included in standard bovine diarrhea-testing panels in many settings, recent detections and isolations from cattle in multiple regions suggest that MRV may represent an under-recognized component of the bovine enteric virome. Importantly, MRV detection in diarrhoeic feces does not by itself establish causality and should be interpreted alongside other major enteric pathogens and relevant clinical and epidemiological information [12,13,14].

Despite increasing reports of MRV in diverse hosts, comparable, site-resolved estimates of MRV positivity in cattle remain limited in south-western China. To address this gap, we conducted a region-wide screening of diarrhoeic cattle in Guangxi between 2023 and 2025 and collected 178 specimens from eight prefectures. We quantified MRV detection by prefecture using L1-targeted RT-PCR and recovered two complete S1 segments to assign serotypes (MRV1 and MRV3) and to place Guangxi sequences within a broader phylogenetic context. These data provide the baseline regional metrics that may support follow-up studies incorporating broader pathogen panels, and help to prioritize targeted sampling and integrated surveillance for bovine diarrheal disease in Guangxi.

## 2. Materials and Methods

### 2.1. Sampling and MRV Detection in Guangxi, China

Between 2023 and 2025, a total of 178 bovine specimens were collected in Guangxi, China, from diarrhoeic cattle. Sampling was case-based and opportunistic. Farms were enrolled through routine veterinary visits and local submissions when diarrheal cases were reported, rather than through a prefecture-stratified design; thus, sample numbers varied among prefectures, according to case availability and field access. Eligible animals were defined by the presence of loose or watery feces at sampling. Specimens were mainly feces, and intestinal contents were collected when available. Samples were placed on ice immediately after collection, transported to the laboratory within 24 h, and stored at −80 °C until processing. Metadata including cattle type, age group, farming pattern, and sampling season are summarized in Table 1. Fecal material and intestinal contents were homogenized in phosphate-buffered saline containing 20% (*v*/*v*) glycerol, vortexed, and clarified by centrifugation at 3000× *g* for 5 min. Viral RNA was extracted from clarified supernatants, using the TIANGEN Magnetic Viral DNA/RNA Kit (DP614; TIANGEN Biotech, Beijing, China) according to the manufacturer’s instructions. RNA was eluted in 50 μL RNase-free water and stored at −80 °C. A no-sample extraction blank was included in each batch. MRV screening was performed by nested RT–PCR, targeting the L1 segment as described previously [14], and primer sequences are listed in Table 2. Approximately 500 ng total RNA was reverse-transcribed in a 20 μL reaction using Takara 6210A at 42 °C for 30 min. Primary PCR was carried out in a 50 μL reaction containing 25 μL 2× Vazyme Green Taq Mix (P131-01), 1.0 μM of each outer primer, and a cDNA template, with nuclease-free water to volume. Cycling conditions were 94 °C for 5 min, followed by 35 cycles of 94 °C for 20 s, 50 °C for 30 s, and 72 °C for 30 s, with a final extension at 72 °C for 10 min (expected 416 bp). Secondary PCR was performed in a 25 μL reaction containing 12.5 μL 2× Vazyme Green Taq Mix, 0.5 μM of each nested primer, and 1 μL of primary product, using the same cycling conditions (expected 344 bp). Amplicons were resolved on 1.5% agarose gels and visualized under UV illumination.

### 2.2. Sequence Alignment and Phylogenetic Analyses

For phylogenetic inference from the L1 segment, nucleotide sequences were aligned with ClustalW (default settings). The best-fit substitution model was selected in MEGA v7.0 (“Find Best DNA/Protein Models, ML”), using the BIC, which identified GTR + G. Maximum-likelihood trees were inferred in MEGA under GTR + G with 1000 bootstrap replicates, and branch support is reported as bootstrap percentages. Tree graphics were formatted with the Chiplot online toolkit. Amino-acid analyses were performed separately for the S1 open reading frame. Deduced S1 proteins were aligned with ClustalW in Jalview v2.11.5.0, and the alignments were used for residue-level assessments to characterize sequence variability and length polymorphisms. Specifically, we examined each aligned position to identify conserved and variable residues, documented substitutions and insertions/deletions (indels), and summarized amino-acid identity between sequences from pairwise comparisons. For σ1, residue-level differences and indels were additionally summarized with respect to the N-terminal, central, and C-terminal regions of the protein (based on reported σ1 domain organization) to facilitate the interpretation of region-specific variation. Accession numbers and metadata (host, collection year, location, source) for all L1 and S1 sequences are provided in Supplementary Table S1. Recombination within the L1 alignment was assessed using RDP v4 (RDP, GENECONV, BootScan, MaxChi, Chimaera, and SiScan methods) with default parameters.

### 2.3. S1 Amplification and Bayesian Temporal Dynamics

We analyzed 239 MRV S1 sequences in total—237 public sequences from NCBI (accessed 25 April 2025) and two new Guangxi sequences generated in this study. Sequences were kept if S1 length was ≥1.3 kb, ambiguous (“N”) sites were ≤5%, and sampling year and country/region were available; exact duplicates and near-identical entries from the same place and year were removed. Putative recombinants flagged by at least two methods were excluded. The temporal signal was checked by regressing the root-to-tip distance against sampling time in TreeTime 2025.4, and clear clock outliers were removed. For the Guangxi samples, total RNA libraries were prepared with random priming and sequenced by second-generation high-throughput sequencing at Sangon Biotech (Shanghai, China) on an Illumina (San Diego, CA, USA) paired-end platform (2 × 150 bp). Raw reads were adapter- and quality-trimmed (Q30), de novo-assembled, and polished by reference-guided mapping to the closest MRV S1. Consensus sequences were called, requiring ≥10× depth and a ≥75% majority base per site; positions failing these thresholds were masked (N). Final consensuses were checked for open reading frame integrity. Time-scaled phylogenies were inferred in BEAST v1.10.4 under GTR + Γ + I, a strict molecular clock, and a Bayesian skyline prior; Markov chain Monte Carlo (MCMC) ran for 200 million states with sampling every 20,000. Convergence was assessed in Tracer v1.7.2, retaining runs with ESS > 200; posterior trees were summarized as an MCC tree in TreeAnnotator v10.5.0 and visualized in FigTree v1.4.4. Substitution rates and node ages are reported as posterior means with 95% highest posterior density (HPD) intervals, and full metadata are provided in Supplementary Table S2.

### 2.4. Statistics Analysis

Univariable associations between MRV detection and categorical variables were evaluated using Pearson’s χ^2^ test or Fisher’s exact test, as appropriate. Crude odds ratios (ORs) with 95% confidence intervals (CIs) were calculated; when a zero cell occurred, the Haldane–Anscombe correction was applied. To account for the small number of positive samples and to reduce small-sample bias in multivariable modeling, we fitted a penalized-likelihood logistic regression model, using Firth’s correction. The outcome was the MRV detection status. The primary exposure was the host group, categorized as water buffalo, native yellow cattle, and Angus cattle. The model adjusted for season, production system, farm scale, and age. Because animals sampled from the same farm are not independent, we used farm-clustered robust standard errors. We reported adjusted ORs with 95% CIs, and for the host group, we additionally reported an overall 2-degree-of-freedom test, assessing the differences across the three categories. All tests were two-sided, with a significance threshold of α = 0.05. Statistical analyses were performed in IBM SPSS Statistics, Version 27.0.1 (IBM Corp., Armonk, NY, USA).

## 3. Results

### 3.1. Regional Detection of MRV in Diarrhoeic Cattle, Guangxi

**Figure 1:**
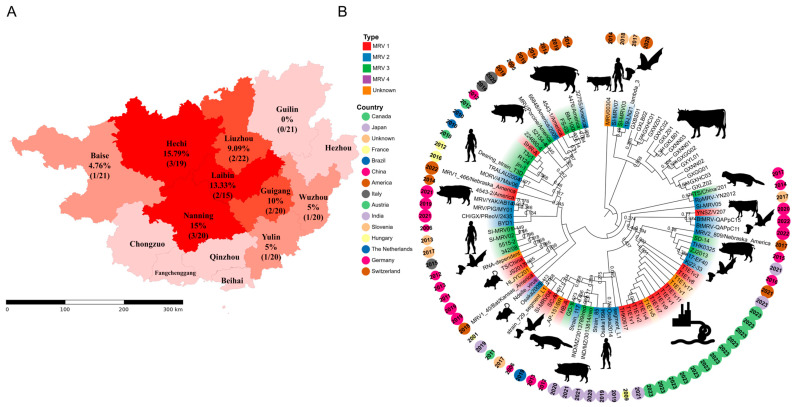
Spatial distribution and L1 phylogeny of bovine MRV in Guangxi. (**A**) MRV positivity by prefecture and sampling locations across Guangxi, based on RT-PCR screening of the L1 fragment; color shading indicates positivity rates. (**B**) Maximum-likelihood phylogram of the partial L1 segment (~344 bp within the RdRp coding region; all sequences trimmed to identical coordinates). The tree was inferred under GTR + G with 1000 bootstrap replicates; branch lengths and a scale bar (substitutions/site) are shown, and a circular layout is used for space. Tip annotations indicate geographic region (color) and host species (symbol), with collection year appended to each label.

From 2023 to 2025, we screened 178 fecal samples from diarrheic cattle across eight prefecture-level cities in Guangxi using RT-PCR, targeting a fragment of the L1 segment. Overall, the MRV positivity was 8.43% (15/178), with clear spatial heterogeneity: Hechi 15.79%, Nanning 15.0%, Laibin 13.33%, Guigang 10.0%, Liuzhou 9.09%, Wuzhou 5.0%, Baise 4.76%, and Guilin 0%. These data indicate ongoing MRV circulation in multiple cattle-producing areas, with higher detection in the northwest/central prefectures (Hechi, Nanning) and no positives identified in Guilin during the study period. While the local sampling intensity may influence point estimates, the aggregate pattern supports heterogeneous MRV activity across the region (Figure 1A and Supplementary Figure S1).

Univariable analyses suggested higher odds of MRV detection in the cool (autumn–winter) season, on non-intensive farms, and in calves, and lower odds in non-Angus cattle; however, only the season reached statistical significance (Table 1). Consistent with this, the three-level host group showed no overall difference in univariable tests (χ^2^ *p* ≈ 0.76). In penalized (Firth) multivariable logistic models adjusting for season, production system, farm scale, and age, the overall host group effect remained non-significant and the category-specific adjusted ORs all had 95% CIs spanning 1.0; the season remained associated with positivity, while the farming pattern, age, and host group were not significant.

Phylogenetic analysis of the partial L1 segment (Figure 1B) resolved all Guangxi MRV-positive sequences into a single, well-supported monophyletic clade. This clade is nested within a broader lineage that includes bovine-derived MRVs from the United States, bat-derived MRV2 from Indonesia, a human MRV2 from Slovenia, and bat-derived MRV3 from Europe. Across the 2023–2025 collection, Guangxi sequences consistently occupy the same branch as these multi-host, multi-region references, with no additional Guangxi lineages observed in the current dataset. Recombination screening of the L1 alignment using RDP v4 (multiple methods with Bonferroni correction) detected no statistically supported recombination events, supporting the suitability of this fragment for coalescent and phylogenetic inference. Collectively, the topology indicates close relatedness at the L1 locus among the Guangxi strains and their nearest reference sequences.

### 3.2. Whole-S1 Phylogeny and Skyline Dynamics

**Figure 2:**
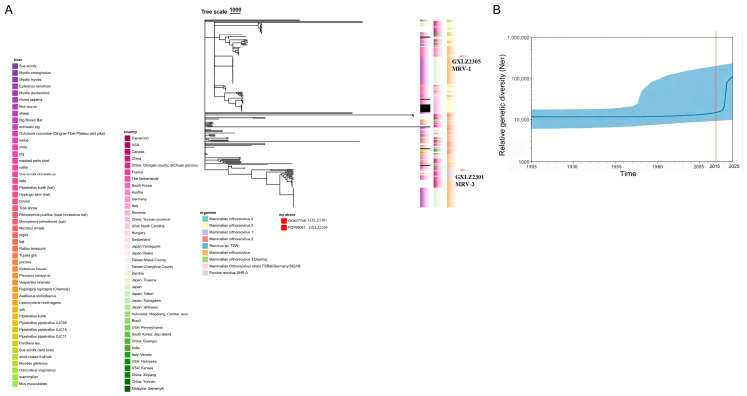
S1 lineage context and descriptive skyline. (**A**) Time-scaled MCC tree for full-length S1 sequences under a strict clock and Bayesian skyline prior. (**B**) Bayesian skyline plot for S1 with the *y*-axis labeled “Relative genetic diversity (Neτ)” (median with 95% HPD band). This figure only shows S1 and is meant as a descriptive view; it does not describe the whole virus.

We obtained two full-length S1 sequences from Guangxi bovine MRVs: GXLZ2301 and GXLZ2305. Using a dataset of 239 S1 sequences (237 public plus the two Guangxi sequences), we performed time-scaled Bayesian analysis to estimate substitution rates and divergence times. In the Bayesian tree (Figure 2A and Figure S2), GXLZ2305 falls within the MRV1 clade, clustering with a Japanese strain (LC613225) and near a Chinese reference (KX263113). GXLZ2301 groups within MRV3 but sits on a distinct internal branch, relative to available references, indicating a separate S1 lineage within MRV3 in this dataset. No additional Guangxi S1 lineages were detected beyond these MRV1- and MRV3-associated placements. The Bayesian skyline (Figure 2B) shows a broadly stable trajectory from ~1905 to 2015, with a recent upward shift (2015–2025) in the median curve; however, the 95% credible intervals are wide, so we interpret this pattern as descriptive rather than conclusive.

### 3.3. σ1 Alignment Reveals Modular Variability and Subtype-Specific Motifs

**Figure 3:**
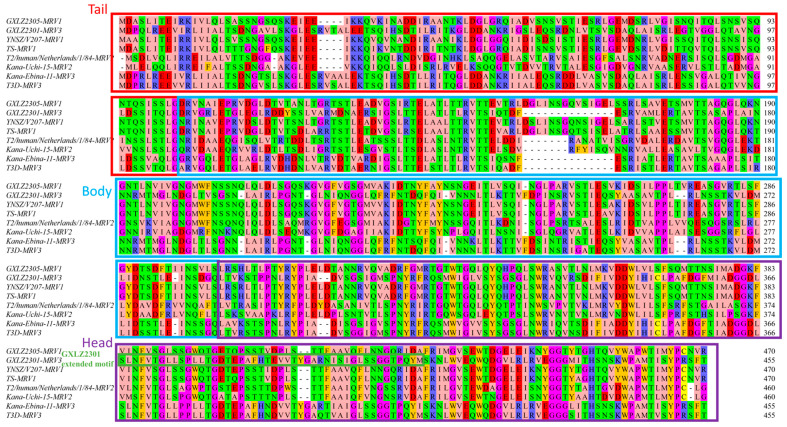
Amino acid variation in σ1 across MRV subtypes. Multiple-sequence alignment of σ1 proteins from the two Guangxi strains (GXLZ2301, MRV3; GXLZ2305, MRV1) and canonical reference sequences representing MRV1–MRV3. The σ1 tail, body, and head regions are indicated, with the tail shown in red, the body in blue, and the head in purple. Conserved segments are evident in the central scaffold and C terminus, whereas the distal head region shows the greatest variability and indel enrichment. A length difference within the head (approximately residues 360–390) is highlighted, with GXLZ2301 showing an extended segment, relative to GXLZ2305 and the MRV1 reference. Residue numbers correspond to the σ1 sequence shown on the right.

Multiple-sequence alignment of σ1 proteins from the two Guangxi strains (GXLZ2301 and GXLZ2305), together with widely used reference sequences representing the three classical MRV serotypes (MRV1–MRV3) revealed a broadly domain-resolved pattern. The N-terminal region (~1–170) appeared relatively indel-prone, with two gap-enriched intervals (~90–110 and ~150–170), followed by a more conserved central segment (~200–320), showing only sporadic substitutions among the sequences examined. The distal portion (~330–470) displayed the greatest variability, including an indel-enriched window around ~360–390, whereas a conserved C-terminal block (~450–470) was largely maintained across the alignment. In this context, the Guangxi MRV3 strain GXLZ2301 showed a longer segment spanning ~360–390 compared with the Guangxi MRV1 strain GXLZ2305 and the MRV1 reference sequence included in the alignment. Given that this comparison relies on a small set of representative, canonical reference sequences (including a single reference per serotype), this length difference is best interpreted as a sequence-level observation from the analyzed strains, rather than a definitive serotype-wide signature. Outside these variable intervals, both Guangxi sequences preserved the conserved central scaffold and C-terminal features observed in the assessed reference sequences (Figure 3).

## 4. Discussion

Our epidemiologic findings are broadly consistent with prior MRV reports and pro-vide a first baseline for Guangxi [15,16]. Positivity seemed higher in the central–western corridor (Hechi–Nanning–Laibin), but uneven sampling means this is only a pattern, not a precise estimate. Season was the only significant factor in univariable analyses, with higher detection in autumn–winter. In Guangxi, cooler temperatures and lower solar radiation in these months likely extend the environmental survival of enteric, non-enveloped MRVs; housing tends to be denser and more indoor, with shared water troughs, bedding, and wetter pens/slurry that increase fecal–oral exposure. Movements linked to markets or slaughter can transiently expand contact networks. Trends toward higher odds in calves and on non-intensive farms, and lower odds in non-Angus cattle, were not significant and had wide confidence intervals, reflecting the modest sample size and limited farm-level data. Without movement records, we do not assign sources or routes; the prefecture-level summary and case-enriched design nevertheless point to practical surveillance targets in this corridor and season, to be refined by larger, balanced surveys [17,18].

Genetically, our data reinforce the known features of MRV evolution while adding new local context. All Guangxi L1 fragments formed a single, well-supported lineage within a broader multi-host background, whereas S1 assigned the two Guangxi strains to different serotypes (MRV1 and MRV3). In the L1 phylogeny, the Guangxi strains clustered with sequences reported from the United States, Slovenia, and Indonesia. Given that L1 is relatively conserved, global sampling remains uneven, and reassortment is common in MRVs, we interpret this pattern as reflecting shared ancestry in a core segment with limited geographic structure, rather than as evidence of recent long-range movement; accordingly, we do not infer routes or directionality from L1 alone. The observed incongruence between L1 and S1 topologies is compatible with, and may plausibly reflect, segment reassortment; however, given that only two complete S1 sequences were obtained and full-genome data are not available here, this should be regarded as a hypothesis supported by limited evidence, rather than a definitive conclusion. Nevertheless, reassortment is well-documented for MRVs, and our findings are consistent with this evolutionary mechanism in Guangxi cattle [19,20].

Recombination screening of the L1 alignment detected no statistically supported events, supporting the use of standard phylogenetic and coalescent models for this fragment [21]. Because we generated only two S1 sequences and public data are uneven across space and time, time-scaled demographic or diffusion analyses would be underpowered and sensitive to sampling bias; we therefore used phylogeny mainly to place the Guangxi strains by lineage/serotype and to give tentative dates [22,23]. We did not perform formal selection tests here and thus do not claim purifying selection [24]. At the protein level, comparison of σ1 suggests a “conserved stalk–variable head” layout, with the MRV3 strain showing an additional motif around 360–390 aa that is absent in the MRV1 strain. As each serotype is represented by a single local sequence, these σ1 differences are pre-liminary signals that warrant testing [25].

These findings have several implications. For surveillance, prioritizing the central–western corridor during cooler months is reasonable, but future work should balance sampling across prefectures and seasons and capture richer metadata on age, breed, production type, and management, so that multivariable models can quantify risk. Linking specimens to movement information (market logs, transport permits, ear-tag data) will allow for direct evaluation of how animal movement shapes prevalence [26,27]. For genomics, generating multi-segment or whole-genome sequences from more positives will let us confirm and map reassortment with models that account for segment exchange, and test for selection with site-wise methods. For function, the σ1 head region—especially the 365–395 aa window—should be examined for receptor/glycan binding and neutralization, and structural work can clarify whether this motif affects the antigenicity. Operationally, conserved regions remain suitable PCR targets, while the variable σ1 head supports including serologic assays that track antigenic change.

Limitations and inference: Sampling was case-based in diarrhoeic cattle and farms were included based on field access and case availability, rather than a pre-specified sampling frame or an a priori sample size calculation. We did not include pen-matched healthy controls. Therefore, our estimates reflect MRV detection among diarrheal cases and should not be interpreted as population prevalence or as evidence that MRV causes diarrhea. Without healthy controls, we cannot quantify background circulation or the strength of association between MRV detection and diarrhea, and future work should incorporate contemporaneous, pen-matched controls with richer metadata to support case–control and multivariable analyses of risk. Laboratory work focused on detection and limited genomic characterization, and we did not perform tissue localization or challenge studies, so causal inference is not possible. We also did not sample wildlife, live-animal markets, or slaughterhouses, so the suggested mixing points and potential reservoirs remain hypotheses and directionality cannot be assigned. Genomic resolution was limited: only two complete S1 sequences were obtained from 15 positives, and most samples yielded L1 fragments rather than full genomes. This restricts our ability to define genome constellations, directly demonstrate reassortment, evaluate mixed infections or within-host variation, test genome-wide selection, or resolve transmission routes versus introductions. Accordingly, we present the phylogenetic patterns as descriptive and avoid source attribution.

## 5. Conclusions

MRV was detected among diarrhoeic cattle in Guangxi, with apparent seasonal variation and the presence of at least two circulating genetic types. Although these observations are based on limited sampling and do not imply causality, they provide preliminary regional data that may inform future surveillance efforts. In particular, consideration of geographic coverage, seasonal timing, basic animal movement information, and routine S1/L1 typing could be useful components of more comprehensive surveillance frameworks. Such approaches may help to refine hypothesis generation regarding MRV circulation and support subsequent targeted investigations into transmission dynamics in cattle populations.

## Figures and Tables

**Table 1:** Correlation analysis of MRV infection ^1^.

Variables (*n* = 178)		Positive	Negative	Comparison (vs. Reference)	*p*-Value	Odds Ratio (OR)	95% Confidence Interval (95% CI)
Age	Calf (101)	10	91	Calf vs. Adult	0.588	1.58	0.52–4.84
	Adult (77)	5	72				
Farming pattern	Intensive (59)	3	56	Non-intensive vs. Intensive	0.391	2.09	0.57–7.73
	Non-intensive (119)	12	107				
Cattle type	Water buffalo (34)	3	31	Non-Angus vs. Angus	0.784	0.73	0.24–2.25
	Native yellow cow (37)	2	35				
	Angus cattle (107)	10	97				
Sampling season	Cool season (90)	13	77	Cool season vs. Warm season	0.0053	7.26	1.59–33.20
	Warm season (88)	2	86				

^1^ We defined a three-level operational host group: water buffalo (*Bubalus bubalis*), native yellow cattle (local *Bos taurus* landraces), and Angus cattle (specialized Bos taurus). Groups were mutually exclusive. Crossbreeds were classified a priori (≥75% Angus as ‘Angus’; otherwise ‘Native/other *Bos taurus*’). Age was classified as calf (≤12 months) vs. adult (>12 months). Production system was intensive (feedlot/pen-feeding or TMR ≥ 90 days) vs. non-intensive (pasture/backyard predominant). Sampling season was cool (October–March) vs. warm (April–September).

**Table 2:** Primer sets for MRV L1 screening and S1 amplification.

Primer Name	Primer Sequences (5′-3′)	Length of PCR Product (bp)
L1-rvF1	GCATCCATTGTAAATGACGAGTCTG	416
L1-rvR1	CTTGAGATTAGCTCTAGCATCTTCTG	
L1-rvF2	GCTAGGCCGATATCGGGAATGCAG	344
L1-rvR2	GTCTCACTATTCACCTTACCAGCAG	

## Data Availability

The data presented in this study are openly available in NCBI [https://www.ncbi.nlm.nih.gov/nuccore/OQ627746; https://www.ncbi.nlm.nih.gov/nuccore/PQ768061/.1/] [OQ627746;PQ768061] (accessed on 12 November 2025).

## Supplementary Materials

The following supporting information can be downloaded at: https://www.mdpi.com/article/10.3390/vetsci13030225/s1, Figure S1: Agarose gel of RT–PCR amplicons for the L1 fragment; Figure S2: Time-scaled S1 MCC tree (descriptive). Table S1: L1 gene sequences used for phylogenetic analysis in this study. Table S2: S1 gene sequences used for phylogenetic analysis in this study.

## Abbreviations

The following abbreviations are used in this manuscript:

MRV	mammalian orthoreoviruses

## References

[1] Torreggiani C., Pupillo G., Garbarino C.A., Rugna G., Prosperi A., Chiapponi C., Luppi A. (2025). Major etiological agents isolated from neonatal calf diarrhea outbreaks in Northern Italy. Pathogens.

[2] Grimwood R.M., Darnley J.A., O’Connell J.P., Hunt H., Taylor H.S., Lawrence K.E., Abbott M.B.W., Jauregui R., Geoghegan J.L. (2025). Oral and faecal viromes of New Zealand calves on pasture with an idiopathic ill-thrift syndrome. Transbound. Emerg. Dis..

[3] Olsen J.E., Svensmark B., Agerskov L., Albrechtsen M., Olsen R.H. (2025). Prevalence and infection characteristics of common pathogens associated with calf diarrhoea in Danish dairy calves. Vet. Microbiol..

[4] Liu Z., Ji S., Chang Q., Wang J., Galon E.M., Xu Y., Yin G., Li J., Gao X., Tian W. (2025). Surveillance of tick-borne viruses in the border regions of the tumen river basin: Co-circulation in ticks and livestock. PLoS Negl. Trop. Dis..

[5] Gao W., Zhang X., Sun M., Han D., Wang J., Li Y., Sanren, Yu L., Gui F., Guo L. (2025). Research of antimicrobial resistance and its associated genes distribution in *Escherichia coli* from diarrheic calves in the Ulagai region of China. Front. Vet. Sci..

[6] Luo Y., Wang Y., Tang W., Wang C., Liu H., Wang X., Xie J., Wang J., Ouyang K., Chen Y. (2024). Isolation and identification of a novel porcine-related recombinant mammalian orthoreovirus type 3 strain from cattle in Guangxi province, China. Front. Microbiol..

[7] Kniert J., Terino D., Eaton H.E., Lin Q.F., Wu S., Strickfaden H., Shmulevitz M. (2025). Spatiotemporal coordination of reovirus peripheral core replication to perinuclear whole virus assembly. PLoS Pathog..

[8] Mazzotta E., Lucchese L., Corro M., Ceglie L., Danesi P., Capello K., Natale A. (2024). Zoonoses in dog and cat shelters in Northeast Italy: Update on emerging, neglected and known zoonotic agents. Front. Vet. Sci..

[9] Zhao D., Li P., Zhang Y., Yu D., Wang T., Zhang K. (2024). First report on the identification and characterization of mammalian orthoreovirus from sheep in China. Microbiol. Spectr..

[10] Li X., Zhao J., Li J., Xiri Y., Liu Z., Zhao Q., Sun Y. (2025). Genome characterization of mammalian orthoreovirus and porcine epidemic diarrhea virus isolated from the same fattening pig. Animals.

[11] DeRuyter E., Williams R.A., Subramaniam K., Lednicky J.A. (2025). Coding complete sequences of the 10 genomic segments of a mammalian orthoreovirus type 3 isolated from a *Blarina peninsulae* shrew. Microbiol. Resour. Announc..

[12] Mao L., Li X., Cai X., Li W., Li J., Yang S., Zhai J., Suolang S., Li B. (2024). First specific detection of mammalian orthoreovirus from goats using TaqMan real-time RT-PCR technology. Vet. Sci..

[13] Wang L., Zheng B., Shen Z., Nath N.D., Li Y., Walsh T., Li Y., Mitchell W.J., He D., Lee J. (2023). Isolation and characterization of mammalian orthoreovirus from bats in the United States. J. Med. Virol..

[14] Leary T.P., Erker J.C., Chalmers M.L., Cruz A.T., Wetzel J.D., Desai S.M., Mushahwar I.K., Dermody T.S. (2002). Detection of mammalian reovirus RNA by using reverse transcription-PCR: Sequence diversity within the lambda3-encoding l1 gene. J. Clin. Microbiol..

[15] Shi K., Li B., Shi Y., Feng S., Yin Y., Long F., Pan Y., Wei Y. (2024). Phylogenetic and evolutionary analysis of porcine epidemic diarrhea virus in Guangxi province, China, during 2020 and 2024. Viruses.

[16] Chen Y., Lin H., Xu S., Nie L., Tang Y., Li X., Zhaxi D., Zhang C., Zhao Q., Zhou E. (2025). Serological and molecular survey of hepatitis e virus in pets in Shaanxi, China. BMC Vet. Res..

[17] Kwon H.J., Kim H.H., Kim H.J., Park J.G., Son K.Y., Jung J., Lee W.S., Cho K.O., Park S.J., Kang M.I. (2012). Detection and molecular characterization of porcine type 3 orthoreoviruses circulating in South Korea. Vet. Microbiol..

[18] Luo Y., Fei L., Yue H., Li S., Ma H., Tang C. (2020). Prevalence and genomic characteristics of a novel reassortment mammalian orthoreovirus type 2 in diarrhea piglets in Sichuan, china. Infect. Genet. Evol..

[19] Yamamoto S.P., Motooka D., Egawa K., Kaida A., Hirai Y., Kubo H., Motomura K., Nakamura S., Iritani N. (2020). Novel human reovirus isolated from children and its long-term circulation with reassortments. Sci. Rep..

[20] Chen J., Meng W., Zeng H., Wang J., Liu S., Jiang Q., Chen Z., Ma Z., Wang Z., Li S. (2024). Epidemiological survey of calf diarrhea related viruses in several areas of Guangdong province. Front. Microbiol..

[21] Flouri T., Huang J., Jiao X., Kapli P., Rannala B., Yang Z. (2022). Bayesian phylogenetic inference using relaxed-clocks and the multispecies coalescent. Mol. Biol. Evol..

[22] Kitamura K., Takagi H., Oka T., Kataoka M., Ueki Y., Sakagami A. (2021). Intertypic reassortment of mammalian orthoreovirus identified in wastewater in Japan. Sci. Rep..

[23] Singh F., Rajukumar K., Senthilkumar D., Venkatesh G., Srivastava D., Kombiah S., Jhade S.K., Singh V.P. (2022). First report on co-isolation and whole-genomic characterisation of mammalian orthorubulavirus 5 and mammalian orthoreovirus type 3 from domestic pigs in India. Arch. Virol..

[24] David Despres G., Lemay G. (2023). Emerging reoviruses: The next pandemic?. Virologie.

[25] Koehler M., Petitjean S., Yang J., Aravamudhan P., Somoulay X., Lo G.C., Poncin M.A., Dumitru A.C., Dermody T.S., Alsteens D. (2021). Reovirus directly engages integrin to recruit clathrin for entry into host cells. Nat. Commun..

[26] Dhar D., Mehanovic S., Moss W., Miller C.L. (2024). Sequences at gene segment termini inclusive of untranslated regions and partial open reading frames play a critical role in mammalian orthoreovirus s gene packaging. PLoS Pathog..

[27] Jandick N.A., Miller C.L. (2023). Creation and characterization of a recombinant mammalian orthoreovirus expressing sigma1 fusion proteins encoding human epidermal growth factor receptor 2 peptides. Virology.

