# Supplementary material for: Prevalence and Genetic Characterization of Mammalian Orthoreoviruses in Diarrheic Cattle from Guangxi, China
Source: Vet Sci. 2026 Feb 27;13(3):225. doi: 10.3390/vetsci13030225 (PMC13030053; doi:10.3390/vetsci13030225)
Supplement: Supplementary file 1 [file vetsci-13-00225-s001.zip › vetsci-4011266-supplementary.pdf]

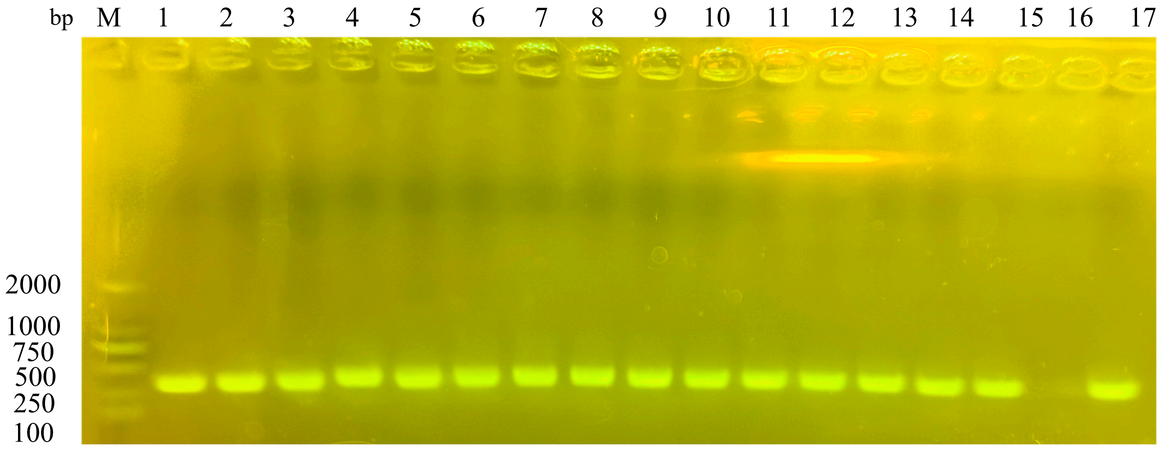

Supplementary Figure S1. Agarose gel of RT–PCR amplicons for the L1 fragment (expected band ~344 bp).

Lanes 1–15: MRV-positive samples; Lane 16: negative control; Lane 17: positive control; M: 2000 bp DNA ladder. Gel: 1.5% agarose; bands were visualized under UV.

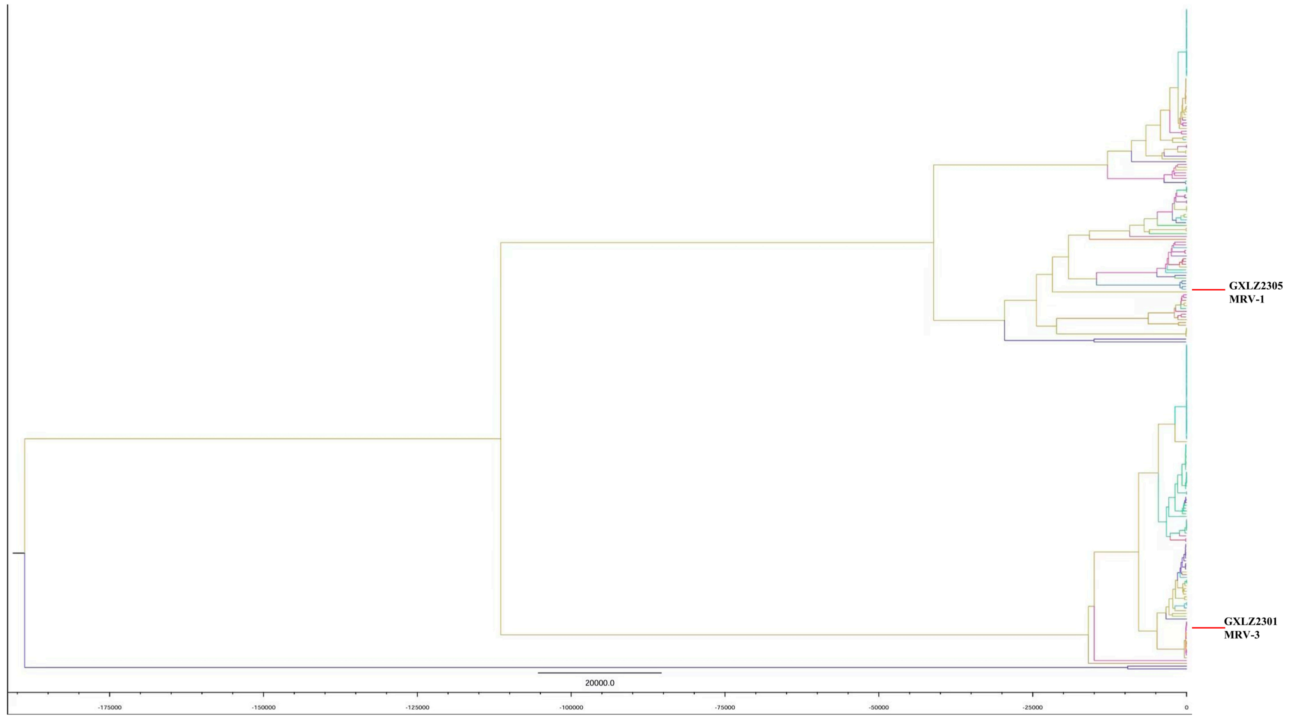

Supplementary Figure S2. Time-scaled S1 MCC tree (descriptive).

BEAST maximum clade credibility tree for full-length S1 (strict clock, Bayesian skyline prior). The x-axis shows model-estimated years before present (software prediction) with a time scale bar. These values are uncertain and not exact calendar dates; use them only to compare relative timing (older left, newer right). Guangxi strains (GXLZ2301, GXLZ2305) are labelled/highlighted. This is an S1-only view for lineage context; we do not draw whole-virus or movement conclusions from this figure

Supplementary Table S1. L1 gene sequences used for phylogenetic analysis in this study.

ID	Type	Country	Date	Isolate	Host
OR074572.1	MRV 1	Canada	2023	T1E1v2	Unknow
LC121916.1	MRV 2	Japan	2016	NO	Unknow
M31058.1	MRV 3	NO	1989	NO	Unknow
HM159613.1	MRV 3	Canada	2010	NO	Homo sapiens
AF368033.1	MRV 4	France	2001	NO	Unknow
OR074666.1	Unknown	Canada	2023	T1E1v12	wastewater
MN022937.1	Unknown	Brazil	2019	NO	Homo sapiens
OR074697.1	Unknown	Canada	2023	T1E1v17	wastewater
OR074656.1	MRV 1	Canada	2023	T1E1v11	wastewater
OR074592.1	MRV 1	Canada	2023	T1E1v4	wastewater
OR074562.1	MRV 1	Canada	2023	T1E1v1	wastewater
MG451071.1	MRV 3	China	2017	NO	Tree shrew
MN233103.1	MRV 3	America	2019	NO	porcine
MN233097.1	MRV 1	America	2019	NO	porcine
MT151659.1	MRV 3	Italy	2020	NO	Sus scrofa domesticus
JN799426.1	Unknown	Austria	2011	NO	piglet
LC476905.1	MRV 2	Japan	2019	NO	Homo sapiens
MN788294.1	Unknown	China	2019	NO	swine
MN788304.1	MRV 1	China	2019	NO	swine
MN233101.1	MRV 2	America	2014	NO	swine
KX343200.1	MRV 3	Italy	2016	NO	swine
MN233098.1	MRV 1	America	2014	NO	porcine
OP169448.1	MRV 2	China	2022	BtMRV-QAPpC15	Pipistrellus pipistrellus XJC15
OR074600.1	Unknown	Canada	2023	T1E1v5	Unknow
MZ516372.1	MRV 3	India	2021	NO	domestic pig
MZ516371.1	MRV 3	India	2021	NO	domestic pig
MN233102.1	MRV 2	America	2005	NO	porcine
MT498602.1	MRV 2	America	2020	19-LN21	Lasionycteris noctivagans
OR074646.1	MRV 1	Canada	2023	T1E1v10	wastewater
M31057.1	MRV 2	NO	1989	NO	Unknow
LC773588.1	MRV 2	Japan	2023	NO	Pteropus vampyrus
LC773578.1	MRV 2	Japan	2023	NO	Pteropus vampyrus
LC773568.1	MRV 2	Japan	2023	NO	Pteropus vampyrus
MG457088.1	MRV 3	Slovenia	2017	NO	Myotis daubentonii

OQ627750.1	MRV 3	China	2023	NO	bovine
KJ676379.1	Unknown	America	2014	NO	bovine
DQ664184.1	MRV 2	China	2006	NO	Unknow
MZ298655.1	MRV 2	China	2021	NO	porcine
MN582420.1	MRV 2	China	2019	NO	pig
MW652775.1	MRV 2	China	2021	NO	yak
OP057385.1	MRV 1	America	2022	NO	Big Brown Bat
OP037832.1	MRV 2	America	2022	NO	Big Brown Bat
KX384846.1	MRV 2	Hungary	2016	NO	Microtus arvalis
JX204738.1	MRV 2	France	2012	NO	Myodes glareolus
JX415466.1	MRV 1	China	2012	NO	porcine
JQ599140.1	MRV 3	Canada	2012	NO	Unknow
M24734.1	MRV 1	NO	1994	NO	Unknow
GU991669.1	MRV 3	The Netherlands	2010	jin-1	Unknow
GU991659.1	MRV 3	The Netherlands	2010	R124	Unknow
MW929746.1	MRV 3	America	2018	NO	pig
KM820754.1	MRV 3	America	2014	FS-03	swine
KM820744.1	MRV 3	America	2014	BM-100	swine
LC705302.1	MRV 3	Japan	2021	NO	Sus scrofa
LC482238.1	MRV 2	Japan	2019	NO	Sus scrofa
LC705292.1	MRV 2	Japan	2020	NO	Sus scrofa
LC705282.1	MRV 2	Japan	2018	NO	Sus scrofa
LC482411.1	MRV 2	Japan	2019	NO	Sus scrofa domesticus
LC482228.1	MRV 2	Japan	2019	NO	Sus scrofa domesticus
MG457108.1	MRV 2	Slovenia	2017	NO	Myotis myotis
MG999576.1	MRV 2	Slovenia	2018	NO	Homo sapiens
KM087105.1	MRV 2	China	2014	NO	Rhinolophus pusillus
OP057407.1	MRV 1	America	2018	NO	Big Brown Bat
KF791261.1	Unknown	China	2013	NO	Rhinolophus pusillus
MG451061.1	MRV 1	China	2011	NO	Tree shrew
JQ412755.1	MRV 3	Germany	2012	NO	bat
JX028412.2	MRV 3	Italy	2015	NO	Pipistrellus kuhlii
KX932029.1	MRV 3	Switzerland	2016	NO	Homo sapiens
MG457078.1	MRV 3	Slovenia	2017	NO	Eptesicus

					serotinus
KF154724.1	MRV 3	Slovenia	2013	NO	Homo sapiens
MW198704.1	MRV 1	China	2020	NO	cattle
OP169447.1	MRV 2	China	2022	BtMRV-QAPpC11	Pipistrellus pipistrellus XJC11
LC579751.1	MRV 3	Japan	2018	NO	Sus scrofa (wild boar)
LC533924.1	MRV 2	Japan	2020	NO	Sus scrofa
JX486057.1	MRV 3	China	2012	GD-1	porcine
MK092964.1	MRV 1	America	2018	OV204	Odocoileus virginianus
LC533914.1	MRV 2	Japan	2020	NO	Sus scrofa
LC533904.1	MRV 2	Japan	2020	NO	Sus scrofa
KY419120.1	MRV 3	China	2016	ZJ2013	pig
KT224504.1	MRV 3	China	2015	NO	mink
LC752173.1	MRV 2	Japan	2023	NO	Vespertilio sinensis
LC613209.1	MRV 2	Japan	2021	NO	Unknow
OP057397.1	MRV 2	America	2017	NO	Big Brown Bat
MW718862.1	MRV 2	America	2021	17-EF40	Eptesicus fuscus
MG457098.1	MRV 1	Slovenia	2017	SI-MRV04	Eptesicus serotinus
KC462149.1	MRV 1	China	2013	HB-A	mink
DQ997719.1	MRV 1	China	2006	NO	Unknow
LC476915.1	MRV 2	Japan	2019	NO	Homo sapiens
LC476895.1	MRV 2	Japan	2019	NO	Homo sapiens
GU196306.1	MRV 2	France	2009	NO	Homo sapiens
LC613219.1	MRV 1	Japan	2021	NO	Unknow
LC705322.1	MRV 3	Japan	2021	NO	Unknow
LC705312.1	MRV 3	Japan	2021	NO	Unknow
OR074636.1	MRV 1	Canada	2023	T1E1v9	wastewater
OR074616.1	MRV 1	Canada	2023	T1E1v7	wastewater
OR074626.1	MRV 1	Canada	2023	T1E1v8	wastewater
OR074582.1	MRV 1	Canada	2023	T1E1v3	wastewater

Supplementary Table S2. S1 gene sequences used for phylogenetic analysis in this study.

ID	Organism	Country	Date	Length	Host	Isolate	Strain
AF368035	Mammalian orthoreovirus 4	Cameroon	4-Apr-25	1423	Mus musculoides	no	no
AY302467	Mammalian orthoreovirus 3	America	31-Oct-06	1385	no	T3/Human/Colorado/1996	no
AY862133	Mammalian orthoreovirus 1	America	16-Jun-05	1413	no	no	no
AY862134	Mammalian orthoreovirus 1	America	16-Jun-05	1416	no	no	no
AY862135	Mammalian orthoreovirus 1	America	16-Jun-05	1413	no	no	no
AY862136	Mammalian orthoreovirus 1	America	16-Jun-05	1413	no	no	no
AY862137	Mammalian orthoreovirus 2	America	16-Jun-05	1383	no	no	no
AY862138	Mammalian orthoreovirus 2	America	16-Jun-05	1383	no	no	no
DQ220017	Reovirus sp. T2W	Canada	15-Sep-06	1435	no	no	T2W
DQ312301	Mammalian orthoreovirus 2	China	18-Apr-06	1437	no	BYD1	no
DQ911244	Mammalian orthoreovirus	China: Qionglai county, SiChuan province	25-Sep-06	1416	no	no	SC-A
EF133	Mamma	Canada	27-Jun-	1309	no	TA	no

509	lian orthoreovirus 3		14			CA46	
EF494441	Mammalian orthoreovirus 3	America	11-Apr-08	1416	no	Dearing	T3D
EF494445	Mammalian orthoreovirus 1	America	11-Apr-08	1463	no	Lang	T1L
EU049603	Mammalian orthoreovirus 2	China	6-Oct-08	1437	no	302I	no
EU049604	Mammalian orthoreovirus 2	China	6-Oct-08	1437	no	302II	no
EU049605	Mammalian orthoreovirus 2	China	6-Oct-08	1437	no	BLD	no
EU049606	Mammalian orthoreovirus 2	China	6-Oct-08	1437	no	BYL	no
EU049607	Mammalian orthoreovirus 2	China	6-Oct-08	1437	no	JP	no
GU196315	Mammalian orthoreovirus 2	France	30-Nov-09	1423	Homo sapiens	no	MRV2Tou05
GU589583	Mammalian orthoreovirus 3	Canada	2-Mar-10	1416	no	no	Abney
GU991665	Mammalian orthoreovirus 3	The Netherlands	1-Nov-12	1416	no	R124	no
GU991675	Mammalian orthoreovirus 3	The Netherlands	1-Nov-12	1416	no	jin-1	no
HM159619	Mammalian orthoreo	America	2-Jan-25	1416	Homo sapiens	Dearing	T3D

	virus 3 Dearing						
JF829213	Mammalian orthoreovirus	South Korea	27-Jun-05	1416	pig	no	KPR110
JF829214	Mammalian orthoreovirus	South Korea	27-Jun-05	1416	pig	no	KPR113
JF829215	Mammalian orthoreovirus	South Korea	27-Jun-05	1416	pig	no	KPR143
JF829216	Mammalian orthoreovirus	South Korea	27-Jun-05	1416	pig	no	KPR146
JF829217	Mammalian orthoreovirus	South Korea	27-Jun-05	1416	pig	no	KPR150
JF829218	Mammalian orthoreovirus	South Korea	27-Jun-05	1416	pig	no	KPR155
JF829219	Mammalian orthoreovirus	South Korea	27-Jun-05	1416	pig	no	KPR157
JF829220	Mammalian orthoreovirus	South Korea	27-Jun-05	1416	pig	no	KPR A
JF829221	Mammalian orthoreovirus	South Korea	27-Jun-05	1416	pig	no	KPR E
JF829222	Mammalian orthoreovirus	South Korea	27-Jun-05	1416	pig	no	KPR G
JN799419	Mammalian orthoreovirus	Austria	20-Jun-05	1436	piglet	no	729
JQ412761	Mammalian Orthoreovirus	Germany	30-Jun-05	1416	bat	no	T3/Bat/Germany/342/08

	strain T3/Bat/Germany/342/08						
JQ599138	Mammalian orthoreovirus 3	Canada	12-Jun-12	1416	no	no	variant T3v2
JQ979271	Mammalian orthoreovirus 3	Italy	2-Apr-13	1416	bats	no	T3/Pipistrellus_khulii/Italy/130366/2011
JQ979272	Mammalian orthoreovirus 3	Italy	2-Apr-13	1416	bats	no	T3/Pipistrellus_Khulii/Italy/5515-2/2012
JQ979273	Mammalian orthoreovirus 3	Italy	2-Apr-13	1416	bats	no	T3/Pipistrellus_Khulii/Italy/5515-14/2012
JQ979274	Mammalian orthoreovirus 3	Italy	2-Apr-13	1416	bats	no	T3/Pipistrellus_khulii/Italy/155012/2011
JQ979275	Mammalian orthoreovirus 3	Italy	2-Apr-13	1416	bats	no	T3/Vespertillio_murinus/Italy/206645-31/2011
JQ979276	Mammalian orthoreovirus 3	Italy	2-Apr-13	1416	bats	no	T3/Tadarida_teniotis/Italy/206645-50/2011
JQ979277	Mammalian orthoreovirus 3	Italy	02013/4/2	1416	bats	no	T3/Tadarida_teniotis/Italy/206645-51/2011
JQ979278	Mammalian orthoreovirus 3	Italy	2-Apr-13	1416	bats	no	T3/Pipistrellus_khulii/Italy/206645-53/2011
JQ979	Mamma	Italy	02013/	1416	bats	no	T3/Pipistr

279	lian orthoreo virus 3		4/2				ellus_khu lii/Italy/2 06645- 54/2011
JQ979 280	Mamma lian orthoreo virus 3	Italy	2-Apr-13	1416	bats	no	T3/Pipistr ellus_khu lii/Italy/2 06645- 56/2011
JQ979 281	Mamma lian orthoreo virus 3	Italy	2-Apr-13	1416	bats	no	T3/Pipistr ellus_khu lii/Italy/2 06645- 57/2011
JQ979 282	Mamma lian orthoreo virus 3	Italy	2-Apr-13	1416	bats	no	T3/Pipistr ellus_khu lii/Italy/2 06645- 58/2011
JQ979 283	Mamma lian orthoreo virus 3	Italy	2-Apr-13	1416	bats	no	T3/Pipistr ellus_khu lii/Italy/2 06645- 60/2011
JQ979 284	Mamma lian orthoreo virus 3	Italy	2-Apr-13	1416	bats	no	T3/Pipistr ellus_Kh ulii/Italy/ 206645- 63/2011
JQ979 285	Mamma lian orthoreo virus 3	Italy	2-Apr-13	1416	bats	no	T3/Pipistr ellus_khu lii/Italy/2 06645- 64/2011
JX204 737	Mamma lian orthoreo virus 2	Germany	8-Apr-04	1453	Myodes glareolus	no	TRALA U2004
JX415 469	Porcine reovirus SHR-A	China	10-May-11	1465	porcine	SHR-A	no
JX486 063	Mamma lian orthoreo virus 3	China	1-Aug-12	1416	porcine	GD-1	no
KC462 155	Mamma lian orthoreo virus	China	4-Jan-13	1462	mink	HB-A	no
KF013	Mamma	China	4-Jan-	1462	mink	no	HB-B

855	lian orthoreo virus 1		13				
KF013 857	Mamma lian orthoreo virus 1	China	4-Jan-13	1462	mink	no	HB-C
KF154 730	Mamma lian orthoreo virus	Slovenia	4-Nov-13	1416	Homo sapiens	SI-MRV01	no
KJ676 385	Mamma lian orthoreo virus	America	20-Feb-14	1466	bovine	no	C/bovine/ Indiana/ MRV003 04/2014
KJ806 994	Mamma lian orthoreo virus 3	The Netherlan ds	2-Nov-14	1416	no	jin-3	no
KM08 7111	Mamma lian orthoreo virus	China: Yunnan province	1-Aug-12	1437	Rhinoloph us pusillus (least horseshoe bat)	RpMR V-YN201 2	no
KM82 0750	Mamma lian orthoreo virus 3	America: North Carolina	6-Jul-05	1416	swine	BM-100	no
KM82 0760	Mamma lian orthoreo virus 3	America: North Carolina	6-Jul-05	1416	swine	FS-03	no
KP208 810	Mamma lian orthoreo virus 3	Canada	24-Jul-02	1368	mammalia n	T3D-S	no
KP208 820	Mamma lian orthoreo virus 3	Canada	7-Aug-03	1368	mammalia n	P4L-12	no
KT224 510	Mamma lian orthoreo virus 3	China	14-Oct-25	1415	mink	no	SD-14
KT900 701	Mamma lian orthoreo virus	Italy	6-Aug-11	1416	bat	BatMR V1-IT2011	no
KU194 672	Mamma lian	Italy	3-Jul-05	1352	Pipistrellu s kuhlii	T3/Pipi strellus	no

	orthoreovirus 2					_kuhlii/Italy/5515-3/2012	
KX263313	Mammalian orthoreovirus 1	China	14-Jun-16	1413	short-nosed fruit bat	B/03	no
KX343206	Mammalian orthoreovirus 3	Italy	15-Feb-25	1393	swine	MRV3/Swine/Italy/224660-4/2015	no
KX384852	Mammalian orthoreovirus	Hungary	28-Jun-05	1433	Microtus arvalis	no	MORV/47Ma/06
KX932035	Mammalian orthoreovirus	Switzerland	15-Nov-25	1416	Homo sapiens	mew716_MRV-3	no
KY419126	Mammalian orthoreovirus 3	China	5-Jul-05	1416	pig	ZJ2013	no
LC121909	Mammalian orthoreovirus 2	Japan:Yamaguchi	3-Jul-05	1425	Panthera leo	no	no
LC476901	Mammalian orthoreovirus 2	Japan:Osaka	16-Jun-05	1401	Homo sapiens	no	Osaka1994
LC476911	Mammalian orthoreovirus 2	Japan:Osaka	27-Jun-05	1402	Homo sapiens	no	Osaka2005
LC476921	Mammalian orthoreovirus 2	Japan:Osaka	6-Jul-05	1402	Homo sapiens	no	Osaka2014
LC482234	Mammalian orthoreovirus 2	Taiwan:Miaoli County	2-Nov-15	1437	Sus scrofa domesticus	no	sR1521
LC482244	Mammalian orthoreovirus 2	Taiwan:Changhua County	13-Nov-15	1433	Sus scrofa domesticus	no	sR1590
LC482	Mamma	Taiwan:C	3-Dec-	1437	Sus scrofa	no	sR1677

417	lian orthoreovirus 2	hanghua County	15		domesticus		
LC533910	Mammalian orthoreovirus	Zambia	10-Jul-05	1433	Sus scrofa	no	Strain 85
LC533920	Mammalian orthoreovirus	Zambia	10-Jul-05	1433	Sus scrofa	no	Strain 96
LC533930	Mammalian orthoreovirus	Zambia	10-Jul-05	1433	Sus scrofa	no	Strain 117
LC579757	Mammalian orthoreovirus 3	Japan: Toyama	7-Nov-18	1416	Sus scrofa (wild boar)	no	MRV-3/Wild boar/Toyama14/2018/JPN
LC613215	Mammalian orthoreovirus 2	Japan	12-Jul-05	1404	no	THK0325	no
LC613225	Mammalian orthoreovirus 1	Japan	12-Jul-05	1374	no	THK0617	no
LC705288	Mammalian orthoreovirus 2	Japan: Tottori	27-Jun-18	1426	Sus scrofa	no	MRV-2/Pig/Totto-MoI6/2018/JPN
LC705298	Mammalian orthoreovirus 2	Japan: Kanagawa	9-Oct-20	1412	Sus scrofa	no	MRV-2/Pig/Kana-Uchi-15/2020/JPN
LC705308	Mammalian orthoreovirus 3	Japan: Ishikawa	24-May-21	1398	Sus scrofa	no	MRV-3/Pig/Ishi-Ueno-10/2021/JPN
LC705318	Mammalian orthoreovirus 3	Japan: Kanagawa	12-Oct-21	1414	Sus scrofa	no	MRV-3/Pig/Kana-Ebina-9/2021/JPN
LC705328	Mammalian	Japan: Kanagaw	12-Oct-21	1416	Sus scrofa	no	MRV-3/Pig/Ka

	orthoreovirus 3	a					na-Ebina-11/2021/JPN
LC752179	Mammalian orthoreovirus	Japan	22-Jul-25	1437	Vespertilio sinensis	Kj22-33	no
LC773574	Mammalian orthoreovirus	Indonesia: Magelang, Central Java	12-May-25	1437	Pteropus vampyrus	MRV2/Bat/Indonesia/IFB12-52/2012	no
LC773584	Mammalian orthoreovirus	Indonesia: Magelang, Central Java	12-May-25	1437	Pteropus vampyrus	MRV2/Bat/Indonesia/IFB12-47/2012	no
LC773594	Mammalian orthoreovirus	Indonesia: Magelang, Central Java	12-May-25	1437	Pteropus vampyrus	MRV2/Bat/Indonesia/IFB12-48/2012	no
LC818341	Mammalian orthoreovirus	Japan	27-Sep-21	1414	Bos taurus	no	MRV/bovine/JPN/HH-1/2021
LC818351	Mammalian orthoreovirus	Japan	27-Sep-21	1445	Bos taurus	no	MRV/bovine/JPN/HH-2/2021
LC818352	Mammalian orthoreovirus	Japan	27-Sep-21	1409	Bos taurus	no	MRV/bovine/JPN/HH-2/2021
LC818365	Mammalian orthoreovirus	Japan	27-Sep-21	1403	Bos taurus	no	MRV/bovine/JPN/HH-3/2021
LC818377	Mammalian orthoreovirus	Japan	27-Sep-21	1448	Bos taurus	no	MRV/bovine/JPN/HH-4/2021
LC818378	Mammalian orthoreovirus	Japan	27-Sep-21	1399	Bos taurus	no	MRV/bovine/JPN/HH-4/2021
LC818	Mamma	Japan	27-Sep-	1409	Bos taurus	no	MRV/bov

390	lian orthoreo virus		21				ine/JPN/ HH- 5/2021
LC818 400	Mamma lian orthoreo virus	Japan	27-Sep- 21	1442	Bos taurus	no	MRV/bov ine/JPN/ HH- 6/2021
LC818 401	Mamma lian orthoreo virus	Japan	27-Sep- 21	1403	Bos taurus	no	MRV/bov ine/JPN/ HH- 6/2021
LC818 412	Mamma lian orthoreo virus	Japan	27-Sep- 21	1412	Bos taurus	no	MRV/bov ine/JPN/ HH- 7/2021
LC818 422	Mamma lian orthoreo virus	Japan	27-Sep- 21	1403	Bos taurus	no	MRV/bov ine/JPN/ HH- 8ko/2021
LC818 432	Mamma lian orthoreo virus	Japan	27-Sep- 21	1437	Bos taurus	no	MRV/bov ine/JPN/ HH- 8oya/202 1
LC818 433	Mamma lian orthoreo virus	Japan	27-Sep- 21	1407	Bos taurus	no	MRV/bov ine/JPN/ HH- 8oya/202 1
LC818 444	Mamma lian orthoreo virus	Japan	27-Sep- 21	1447	Bos taurus	no	MRV/bov ine/JPN/ HH- 9ko/2021
LC818 445	Mamma lian orthoreo virus	Japan	27-Sep- 21	1408	Bos taurus	no	MRV/bov ine/JPN/ HH- 9ko/2021
LC818 457	Mamma lian orthoreo virus	Japan	27-Sep- 21	1448	Bos taurus	no	MRV/bov ine/JPN/ HH- 9oya/202 1
LC818 458	Mamma lian orthoreo virus	Japan	27-Sep- 21	1401	Bos taurus	no	MRV/bov ine/JPN/ HH- 9oya/202 1
LC818 470	Mamma lian orthoreo	Japan	28-Sep- 21	1410	Bos taurus	no	MRV/bov ine/JPN/ HH-

	virus						10ko/2021
LC818480	Mammalian orthoreovirus	Japan	28-Sep-21	1440	Bos taurus	no	MRV/bovine/JPN/HH-10oya/2021
LC818481	Mammalian orthoreovirus	Japan	28-Sep-21	1414	Bos taurus	no	MRV/bovine/JPN/HH-10oya/2021
LC818493	Mammalian orthoreovirus	Japan	28-Sep-21	1447	Bos taurus	no	MRV/bovine/JPN/HH-11ko/2021
LC818494	Mammalian orthoreovirus	Japan	28-Sep-21	1416	Bos taurus	no	MRV/bovine/JPN/HH-11ko/2021
LC818507	Mammalian orthoreovirus	Japan	28-Sep-21	1448	Bos taurus	no	MRV/bovine/JPN/HH-11oya/2021
LC818508	Mammalian orthoreovirus	Japan	28-Sep-21	1396	Bos taurus	no	MRV/bovine/JPN/HH-11oya/2021
LC818521	Mammalian orthoreovirus	Japan	28-Sep-21	1448	Bos taurus	no	MRV/bovine/JPN/HH-12/2021
LC818531	Mammalian orthoreovirus	Japan	28-Sep-21	1402	Bos taurus	no	MRV/bovine/JPN/HH-13/2021
LC818541	Mammalian orthoreovirus	Japan	29-Sep-21	1402	Bos taurus	no	MRV/bovine/JPN/HH-14/2021
LC818551	Mammalian orthoreovirus	Japan	29-Sep-21	1414	Bos taurus	no	MRV/bovine/JPN/HH-15/2021
LC818561	Mammalian	Japan	29-Sep-21	1448	Bos taurus	no	MRV/bovine/JPN/

	orthoreo virus						HH-16/2021
LC818 562	Mamma lian orthoreo virus	Japan	29-Sep-21	1410	Bos taurus	no	MRV/bov ine/JPN/ HH-16/2021
LC818 573	Mamma lian orthoreo virus	Japan	29-Sep-21	1435	Bos taurus	no	MRV/bov ine/JPN/ HH-17/2021
LC818 574	Mamma lian orthoreo virus	Japan	29-Sep-21	1398	Bos taurus	no	MRV/bov ine/JPN/ HH-17/2021
LC818 585	Mamma lian orthoreo virus	Japan	29-Sep-21	1445	Bos taurus	no	MRV/bov ine/JPN/ HH-18/2021
LC818 586	Mamma lian orthoreo virus	Japan	29-Sep-21	1403	Bos taurus	no	MRV/bov ine/JPN/ HH-18/2021
LC818 598	Mamma lian orthoreo virus	Japan	29-Sep-21	1448	Bos taurus	no	MRV/bov ine/JPN/ HH-19/2021
LC818 609	Mamma lian orthoreo virus	Japan	29-Sep-21	1447	Bos taurus	no	MRV/bov ine/JPN/ HH-20/2021
LC818 610	Mamma lian orthoreo virus	Japan	29-Sep-21	1403	Bos taurus	no	MRV/bov ine/JPN/ HH-20/2021
LC818 621	Mamma lian orthoreo virus	Japan	29-Sep-21	1410	Bos taurus	no	MRV/bov ine/JPN/ HH-21/2021
LC818 633	Mamma lian orthoreo virus	Japan	29-Sep-21	1443	Bos taurus	no	MRV/bov ine/JPN/ HH-22/2021
LC818 634	Mamma lian orthoreo virus	Japan	29-Sep-21	1402	Bos taurus	no	MRV/bov ine/JPN/ HH-22/2021
LC818 645	Mamma lian orthoreo virus	Japan	29-Sep-21	1410	Bos taurus	no	MRV/bov ine/JPN/ HH-23/2021

LC818655	Mammalian orthoreovirus	Japan	29-Sep-21	1447	Bos taurus	no	MRV/bovine/JPN/HH-24/2021
LC818656	Mammalian orthoreovirus	Japan	29-Sep-21	1408	Bos taurus	no	MRV/bovine/JPN/HH-24/2021
LC818667	Mammalian orthoreovirus	Japan	29-Sep-21	1445	Bos taurus	no	MRV/bovine/JPN/HH-25/2021
LC818668	Mammalian orthoreovirus	Japan	29-Sep-21	1405	Bos taurus	no	MRV/bovine/JPN/HH-25/2021
LC818679	Mammalian orthoreovirus	Japan	29-Sep-21	1447	Bos taurus	no	MRV/bovine/JPN/HH-26/2021
LC818689	Mammalian orthoreovirus	Japan	29-Sep-21	1449	Bos taurus	no	MRV/bovine/JPN/HH-27/2021
LC818699	Mammalian orthoreovirus	Japan	29-Sep-21	1453	Bos taurus	no	MRV/bovine/JPN/HH-28/2021
LC818709	Mammalian orthoreovirus	Japan	29-Sep-21	1423	Bos taurus	no	MRV/bovine/JPN/HH-29/2021
LC818710	Mammalian orthoreovirus	Japan	29-Sep-21	1409	Bos taurus	no	MRV/bovine/JPN/HH-29/2021
LC818721	Mammalian orthoreovirus	Japan	29-Sep-21	1414	Bos taurus	no	MRV/bovine/JPN/HH-30/2021
LC818731	Mammalian orthoreovirus	Japan	29-Sep-21	1445	Bos taurus	no	MRV/bovine/JPN/HH-31/2021
LC818732	Mammalian orthoreovirus	Japan	29-Sep-21	1412	Bos taurus	no	MRV/bovine/JPN/HH-31/2021
LC818744	Mammalian	Japan	29-Sep-21	1405	Bos taurus	no	MRV/bovine/JPN/

	orthoreovirus						HH-32ko/2021
LC818755	Mammalian orthoreovirus	Japan	29-Sep-21	1406	Bos taurus	no	MRV/bovine/JPN/HH-32oya/2021
LC818765	Mammalian orthoreovirus	Japan	28-Sep-21	1443	Bos taurus	no	MRV/bovine/JPN/HH-100ko/2021
LC818775	Mammalian orthoreovirus	Japan	28-Sep-21	1448	Bos taurus	no	MRV/bovine/JPN/HH-100oya/2021
LC818776	Mammalian orthoreovirus	Japan	28-Sep-21	1402	Bos taurus	no	MRV/bovine/JPN/HH-100oya/2021
LC818789	Mammalian orthoreovirus	Japan	29-Sep-21	1402	Bos taurus	no	MRV/bovine/JPN/HH-101ko/2021
LC818799	Mammalian orthoreovirus	Japan	28-Sep-21	1408	Bos taurus	no	MRV/bovine/JPN/HH-102/2021
MG451067	Mammalian orthoreovirus	China	19-Aug-18	1481	Tree shrew	MRV1/TS/China/2011	no
MG451077	Mammalian orthoreovirus	China	19-Aug-18	1346	Tree shrew	MRV3/TS/China/2012	no
MG457084	Mammalian orthoreovirus	Slovenia	2-Jul-05	1416	Eptesicus serotinus	SI-MRV02	no
MG457094	Mammalian orthoreovirus	Slovenia	4-Jul-05	1463	Myotis daubentonii	SI-MRV03	no
MG457104	Mammalian	Slovenia	1-Jul-05	1454	Eptesicus serotinus	SI-MRV04	no

	orthoreo virus						
MG45 7114	Mamma lian orthoreo virus	Slovenia	30-Jun-05	1433	Myotis myotis	SI-MRV05	no
MG45 7124	Mamma lian orthoreo virus	Slovenia	1-Jul-05	1417	Myotis emarginat us	SI-MRV06	no
MG99 9582	Mamma lian orthoreo virus	Slovenia	9-Jul-05	1440	Homo sapiens	SI-MRV07	no
MG99 9583	Mamma lian orthoreo virus	Slovenia	9-Jul-05	1323	Homo sapiens	SI-MRV07	no
MH82 2896	Mamma lian orthoreo virus 3	America	11-Sep-18	1416	no	no	T3SA+
MH93 3775	Mamma lian orthoreo virus 2	Cameroon	6-Jul-05	1417	Homo sapiens	T2/CM R/Hum an/CM R-HP55/2 014 segmen t S1	no
MK09 2970	Mamma lian orthoreo virus 2	America	8-Jul-05	1386	Odocoileu s virginianu s	OV204	no
MK24 6422	Mamma lian orthoreo virus 3	Canada	09-JAN-2019	1368	no	no	T3 Dearing VeroAV
MK27 9735	Mamma lian orthoreo virus 1	China	9-Jul-05	1453	porcine	no	MRV1/pi g-wt/CHN/ F19/2017 08
MK40 8605	Mamma lian orthoreo virus	Italy	1-Jul-05	1392	Rupicapra rupicapra (Chamois)	MRV-3 chamoi s 84407 Italy 2009	no
MN02 2930	Mamma lian	Brazil	23-Dec-19	1326	Homo sapiens	no	AP-151/BR

	orthoreo virus						
MN23 3091	Mamma lian orthoreo virus	America	6-Jul-05	1409	porcine	no	MRV2/Pi g/32755/ America/ 2014
MN23 3092	Mamma lian orthoreo virus	America	27-Jun-05	1409	porcine	no	MRV2/Pi g/66848/ America/ 2005
MN23 3093	Mamma lian orthoreo virus	America	6-Jul-05	1484	porcine	no	MRV2/Pi g/4560-2/Americ a/2014
MN23 3094	Mamma lian orthoreo virus	America	6-Jul-05	1419	porcine	no	MRV2/Pi g/4560-1/Americ a/2014
MN23 3095	Mamma lian orthoreo virus	America	6-Jul-05	1474	porcine	no	MRV1/Pi g/4543/A merica/20 14
MN23 3096	Mamma lian orthoreo virus	America	6-Jul-05	1399	porcine	no	MRV3/Pi g/4476/A merica/20 14
MN58 2426	Mamma lian orthoreo virus	China	18-Oct-25	1434	pig	MRV/P IG/MY 01/201 8	no
MN78 8300	Mamma lian orthoreo virus	China	9-Jul-05	1461	swine	no	HLJYC2 017
MN78 8310	Mamma lian orthoreo virus 1	China	9-Jul-05	1461	swine	no	JS2017
MT151 671	Mamma lian orthoreo virus 2	Italy	10-Jul-05	1382	Sus scrofa domesticu s	MRV2/ Swine/I taly/90 178-3/2018 _S1	no
MT151 672	Mamma lian orthoreo virus 3	Italy	8-Jul-05	1353	Sus scrofa domesticu s	MRV3/ Swine/I taly/52 154-4/2016 _S1	no

MT498608	Mammalian orthoreovirus	America: Pennsylvania	11-Jul-05	1434	Lasionycteris noctivagans	19-LN21	no
MT518190	Mammalian orthoreovirus	Slovenia	3-Jul-05	1439	Homo sapiens	SI-MRV08	no
MT518191	Mammalian orthoreovirus	Slovenia	3-Jul-05	1326	Homo sapiens	SI-MRV08	no
MW149479	Mammalian orthoreovirus	China	10-Jul-05	1434	yak	no	MRV/YAK/AB14/2018
MW198710	Mammalian orthoreovirus 1	China	8-Jul-05	1462	cattle	V207S1	YNSZ/V207/2016
MW582628	Mammalian orthoreovirus	South Korea: Jeju Island	19-Jan-25	1460	Miniopterus schreibersii (bat)	BatMRV/B19-02	no
MW718868	Mammalian orthoreovirus	America: Pennsylvania	9-Jul-05	1434	Eptesicus fuscus	17-EF40	no
MW929752	Mammalian orthoreovirus	America	10-Jul-05	1456	pig	MRV/Porcine/America/2018	no
MZ298661	Mammalian orthoreovirus	China: Guangxi	26-Dec-18	1428	porcine	CH/GX/PReoV/2435/2018	no
MZ541851	Mammalian orthoreovirus	India	17-Mar-25	1410	domestic pig	IND/MZ/3013789/reo	no
MZ541852	Mammalian orthoreovirus	India	17-Mar-25	1405	domestic pig	IND/MZ/3013814/reo	no
MZ687791	Mammalian orthoreovirus	Italy: Veneto	7-Feb-18	1349	swine	no	18DIAPD90114/2
NC_013231	Mammalian	China	26-Jun-05	1368	masked palm civet	MPC/04	no

	orthoreo virus 3						
NC_077844	Mammalian orthoreovirus 3 Dearing	America	2-Jan-25	1416	Homo sapiens	Dearing	T3D
OP019320	Mammalian orthoreovirus 2	America: Nebraska	8-Sep-17	1433	Big Brown Bat	no	MRV2 115/Bat/ Nebraska America/ 2017
OP057378	Mammalian orthoreovirus	America: Nebraska	28-Nov-17	1457	Big Brown Bat	no	MRV1 466/Bat/ Nebraska America/ 2017
OP057390	Mammalian orthoreovirus	America: Nebraska	8-Jun-17	1434	Big Brown Bat	no	MRV2 809/Bat/ Nebraska America/ 2017
OP057400	Mammalian orthoreovirus	America: Kansas	13-Dec-18	1461	Big Brown Bat	no	MRV1 40/Bat/Kansas America/ 2018
OP169465	Mammalian orthoreovirus 2	China: Xinjiang	16-Jul-16	1375	Pipistrellus pipistrellus XJC11	BtMRV-QAPpC11	no
OP169466	Mammalian orthoreovirus 2	China: Xinjiang	16-Jul-16	1422	Pipistrellus pipistrellus XJC15	BtMRV-QAPpC15	no
OP169467	Mammalian orthoreovirus 2	China: Xinjiang	16-Jul-16	1431	Pipistrellus pipistrellus XJC66	BtMRV-QAPpC66	no
OP963630	Mammalian orthoreovirus	China: Yunnan	9-Jul-05	1437	Aselliscus stoliczkanus	no	Bat/2017/ S17BSBatR47
OR074535	Mammalian orthoreovirus	Canada	4-Jul-05	1471	no	T2E2	no
OR074545	Mammalian orthoreo	Canada	4-Jul-05	1380	no	T2E3	no

	virus						
OR074565	Mammalian orthoreovirus	Canada	4-Jul-05	1379	no	T1E1v2	no
OR074585	Mammalian orthoreovirus	Canada	4-Jul-05	1434	no	T1E1v4	no
OR074595	Mammalian orthoreovirus	Canada	4-Jul-05	1377	no	T1E1v5	no
OR074609	Mammalian orthoreovirus	Canada	4-Jul-05	1381	no	T1E1v7	no
OR074619	Mammalian orthoreovirus	Canada	4-Jul-05	1474	no	T1E1v8	no
OR074629	Mammalian orthoreovirus	Canada	4-Jul-05	1438	no	T1E1v9	no
OR074659	Mammalian orthoreovirus	Canada	4-Jul-05	1372	no	T1E1v12	no
OR074680	Mammalian orthoreovirus	Canada	4-Jul-05	1433	no	T1E1v16	no
OR074690	Mammalian orthoreovirus	Canada	4-Jul-05	1407	no	T1E1v17	no
OR074717	Mammalian orthoreovirus	Canada	4-Jul-05	1448	no	T1E1v20	no
OR339562	Mammalian orthoreovirus 3	Italy	11-Jul-05	1374	Hypsugo savii (bat)	no	127044-18
OR339563	Mammalian orthoreovirus 3	Italy	11-Jul-05	1374	Hypsugo savii (bat)	no	127044-19
OR339	Mamma	Italy	11-Jul-	1374	Hypsugo	no	127044-

564	lian orthoreovirus 3		05		savii (bat)		23
OR339565	Mammalian orthoreovirus 3	Italy	11-Jul-05	1374	Pipistrellus kuhlii (bat)	no	127044-39
OR339566	Mammalian orthoreovirus 3	Italy	11-Jul-05	1374	Hypsugo savii (bat)	no	127044-47
OR339567	Mammalian orthoreovirus 3	Italy	11-Jul-05	1374	Pipistrellus kuhlii (bat)	no	127044-50
OR339568	Mammalian orthoreovirus 3	Italy	11-Jul-05	1374	Pipistrellus kuhlii (bat)	no	127044-51
OR339569	Mammalian orthoreovirus 3	Italy	11-Jul-05	1374	Pipistrellus kuhlii (bat)	no	127044-53
OR339570	Mammalian orthoreovirus 3	Italy	11-Jul-05	1374	Hypsugo savii (bat)	no	127044-68
OR339571	Mammalian orthoreovirus 3	Italy	11-Jul-05	1374	Pipistrellus kuhlii (bat)	no	127044-75
OR344327	Mammalian orthoreovirus	China	19-Dec-25	1456	Ochotona curzoniae (Qinghai-Tibet Plateau wild pika)	Pika/MRV/GCCDC7/2019	no
OR392823	Mammalian orthoreovirus 3	China	13-Dec-19	1368	Sus scrofa	HN2019-01	no
PP228845	Mammalian orthoreovirus 3	China	14-Jul-05	1416	porcine	no	MRV3/CHN/GXLB0237/2022
PP235846	Mammalian orthoreovirus	China	20-Apr-23	1466	sheep	XJ23	no
PP942	Mamma	China	11-Apr-	1413	sheep	no	sheep/SY

562	lian orthoreo virus		23				13
PQ306 476	Mamma lian orthoreo virus	China	20-Sep-25	1416	domestic pig	HNU-XXS-2020	no
PQ442 206	Mamma lian orthoreo virus	Malaysia: Semenyih	23-Jan-25	1400	Tupaia glis	MRV1 UNM	24MYSE L
PQ678 210	Mamma lian orthoreo virus	China: Yunnan	14-Jul-05	1368	Rattus tanezumi	QJZY-YNSC2 33	no
PQ768 061	Mamma lian orthoreo virus	China	16-May-23	1462	bovine	no	MRV1/G XLZ2305 /Bovine/ CHN
PV005 841	Mamma lian orthoreo virus	South Korea	24-Jun-25	1386	bat	batMR V2/KV RI1/KO REA/2 024	no
X0116 1	Mamma lian orthoreo virus 3 Dearing	America	19-May-95	1416	no	no	no
